# Promoter cloning and activities analysis of *JmLFY*, a key gene for flowering in *Juglans mandshurica*

**DOI:** 10.3389/fpls.2023.1243030

**Published:** 2023-10-12

**Authors:** Lijie Zhang, Jingqi Fu, Tianyi Dong, Mengmeng Zhang, Jingwen Wu, Chunping Liu

**Affiliations:** ^1^ Key Laboratory of Forest Tree Genetics, Breeding and Cultivation of Liaoning Province, Shenyang Agricultural University, Shenyang, China; ^2^ Key Laboratory of Silviculture of Liaoning Province, Shenyang Agricultural University, Shenyang, China; ^3^ Key Laboratory of Silviculture of Liaoning Province , Shenyang, China

**Keywords:** Manchurian walnut, JmLFY, promoter, functional analysis, genetic transformation, transient transformation, yeast one hybrid

## Abstract

*Juglans mandshurica* (Manchurian walnut) is a precious timber and woody grain and oil species in Northeast China. The heterodichogamous characteristic phenomenon resulted in the non-synchronous flowering and development of male and female flowers, which limited the mating and the yield and quality of fruits. *LFY* is a core gene in the flowering regulatory networks, which has been cloned in *J. mandshurica*, and the function has also been verified preliminarily. In this study, the *JmLFY* promoter sequence with different lengths of 5′-deletion (pLFY1-pLFY6) were cloned and conducted bioinformatics analysis, the promoter activities were analyzed by detecting their driving activity to GUS gene in the tobacco plants that transformed with different promoter sequence stably or transiently. After that, the interaction between JmSOC1 and *JmLFY* gene promoter was also analyzed via yeast single-hybrid. The results showed that the promoter sequence contains core cis-acting elements essential for eukaryotic promoters, hormone response elements, defense- and stress-responsive elements, flowering-related elements, etc. Transgenic tobacco plants with *pLFY1* were obtained by *Agrobacterium* infection using the pCAMBIA1301 expression vector, and the GUS gene driven by the *JmLFY* promoter was detected to express in the leaf, stem, flower, and root of the transformed tobacco plant, which indicated that the obtained *JmLFY* promoter had driving activity. GUS histochemical staining and enzyme activity detection showed that promoter fragments with different lengths had promoter activity and could respond to the induction of long photoperiod, low temperature, salicylic acid (SA), IAA, GA3, and methyl jasmonate (MeJA). The core regulatory region of *JmLFY* gene promoter in *J. mandshurica* was between −657 bp and −1,904 bp. Point-to-point validation of yeast single-hybrid confirmed the interaction between JmSOC1 and *JmLFY* gene promoter, which indicated that *JmLFY* gene is the downstream target of JmSOC1. These results reveal relevant factors affecting *JmLFY* gene expression and clarify the molecular mechanism of *JmLFY* gene regulation in the flower developmental partially, which will provide a theoretical basis for regulating the flowering time by regulating *JmLFY* gene expression in *J. mandshurica*.

## 1 Introduction

Flowering is an important process in the plant life cycle, which means the end of childhood life and the plants begin reproductive growth (Zhang and Liu, 2003; Zhang et al., 2019a; Zhang et al., 2019b). The transitions from vegetative growth to reproductive growth were affected by the combination of internal factors and external environmental factors (Aidyn et al., 2002; Zeng et al., 2018), such as age, photoperiod, vernalization, autonomous pathway, and gibberellin pathway (Martina et al., 2015; Jian et al., 2019; Jin et al., 2019; Liu et al., 2021). Thus, the flower development and flower bud differentiation processes in plants are regulated by complex gene regulatory networks (Wang et al., 2004).

*LFY* has a core position in the flowering regulatory networks (Gordon and Caroline, 2002; Liu et al., 2009; Zhao et al., 2020), which participate in several pathways mentioned above, and plays crucial roles in promoting the formation of floral primordia, maintaining floral meristem function and floral initiation, and preventing the reversal of floral meristem (Shannon and Meeks-Wagner, 1993; Mandel and Yanofsky, 1995; Yanofsky, 1995; Chen et al., 1997; Alvarez-Buylla et al., 2006; He et al., 2018). The overexpression of *LFY* genes promoted early flowering and supplemented the phenotypic defects of *lfy* mutant partially (Weigel et al., 1993; He et al., 2000; Ahearn et al., 2001; Peña et al., 2001; Cai et al., 2023), which demonstrates the important role of *LFY* genes in flowering regulation further.

*Juglans mandshurica* is a precious timber and woody grain and oil species in Northeast China, which has important economic, nutritional, and medicinal values. As a monoecious species, we found that the heterodichogamous characteristics are a common phenomenon in *J. mandshurica* in the previous investigation of the reproductive phenological characteristics of the species (Guo, 2020; Qin et al., 2021), which resulted in the non-synchronous flowering and development of male and female flowers and then limited the mating and the yield and quality of fruits. Therefore, it is necessary to solve the bottleneck problem of low fruit yield caused by the inconsistent development of male and female flowers. We have cloned the *JmLFY* from *J. mandshurica* successfully (Song, 2019; Liu et al., 2022), and the genetic transformation and function verification studies were also conducted by transformed *JmLFY* into *Arabidopsis* (Cai, 2022). Overexpression of *JmLFY* gene in *Arabidopsis* inhibited vegetative growth, promoted reproductive growth, and supplemented the phenotypic defects of *lfy* mutant partially.

Promoters are able to determine the expression level of genes and occupy an important role in the regulation of gene transcription. In this study, we cloned the promoter of *JmLFY* genes and predicted the *cis*-acting elements and active sites on promoters using online bioinformatics software. In order to identify factors affecting the expression of *JmLFY* genes, we constructed a series of plant expression vectors using different lengths of the promoter with 5′-deletion and verified the activities of the promoter. Further validation of interaction between JmSOC1 and the *JmLFY* promoter was also conducted by point-to-point validation of yeast single-hybrid. The results will reveal relevant factors affecting *JmLFY* gene expression and clarify the molecular mechanism of *JmLFY* gene regulation in the flower developmental partially, which will provide a theoretical basis for regulating the flowering time by regulating *JmLFY* gene expression in *J. mandshurica*.

## 2 Materials and methods

### 2.1 *JmLFY* promoter cloning and bioinformatics analysis

DNA extraction from leaves of *J. mandshurica* was conducted as described by Song (2019). According to the obtained *JmLFY* gene sequence (GenBank Accession No.: KX364241), an upstream non-coding nucleotide sequence (NC_049909.1) from *Juglans regia* genomic DNA was selected for *JmLFY* promoter cloning (**Table 1**). PCR amplification (the reaction system included LA Taq Mix 12.5 µL, DNA 1 µL, LFY-P-F and LFY-P-R (10 µmol/L) 1 µL, and ddH_2_O 9.5 µL). The reaction procedure was as follows: 94°C for 5 min; 35 cycles of 94°C for 30 s, 58°C for 1 min, 72°C for 2 min; and 72°C for 7 min; the product was detected by 1% agarose gel electrophoresis and then recovered using the MiniBEST Agarose Gel DNA Extraction Kit version 4.0 (Takara, Dalian, China). The recovered product was then ligated to the pMD-19-T Vector (Takara) according to the instructions. The recombined vector was then transformed into *Escherichia coli* DH5α competent cells, the positive clones selected by 50 mg/L of ampicillin (Amp) were used for PCR amplification and then sequenced, and the correct recombined vector confirmed by sequencing was named pMD19-T-pLFY.

**Table 1:** Primer sequences for cloning and functional analysis of *JmLFY* promoter of *Juglans mandshurica*.

Primers	Sequences (5′–3′)	Usage
*LFY*-P-F	AGGGATTTATGTTCTACTTGGC	*JmLFY* promoter cloning
*LFY*-P-R	ACCATGGGGTTCGGAGGCGGGA	
*pLFY*-F1	CCAAGCTTAGGGATTTATGTTCTACTTGGC	*JmLFY* promoter vector construction
*pLFY*-F2	CGCCAAGCTTCAGCAGATGTGTTTCTATTTCTG	
*pLFY*-F3	CGCCAAGCTTAAGGGTTCACTCCCCGAGTACG	
*pLFY*-F4	CGCCAAGCTTCTACTTCTTGGGCATGAAAGCA	
*pLFY*-F5	CGCCAAGCTTCAAGTCATAATTTCAATTTTTAT	
*pLFY*-F6	CGCCAAGCTTCTTTTCCTTGGGAGAAAAAGTT	
*pLFY*-R1	CTCAGATCTACCATGGGGTTCGGAGGCGGGA	
*ADSOC1*-F	GAGTGGCCATTATGGCCCATGTGTGTTTGCTGTCATAG	pGADT7-Rec2-JmSOC1 prey vector construction
*ADSOC1*-R	GCCGACATGTTTTTTCCCTCAATTCTGTGGGAGGCGCT	
*HISpLFY-*F	CGGAATTCAGGGATTTATGTTCTACTTGGC	pHIS2-pLFY bait vector construction
*HISpLFY-*R	CGACGCGTACCATGGGGTTCGGAGGCGGGA	
*GUS-*F	GCATTCAGTCTGGATCGCGA	*GUS* gene detection
*GUS-*R	TCACCGAAGTTCATGCCAGTCC	
*qGUS-*F	TACCGTACCTCGCATTACCC	*GUS* gene quantification
*qGUS-*R	CTGTAAGTGCGCTTGCTGAG	
*qL25*-F	GCTAAGGTTGCCAAGGCTGTC	
*qL25*-R	TAAGGTATTGACTTTCTTTGTCTGA	

The obtained promoter sequence was aligned by DNAMAN6.0, and then regulatory elements were predicted and analyzed using online analysis software Plant CARE (http://bioinformatics.psb.ugent.be/webtools/plantcare/html/) and PLACE (https://www.dna.affrc.go.jp/PLACE/).

### 2.2 Expression vector construction

**Figure 1:**
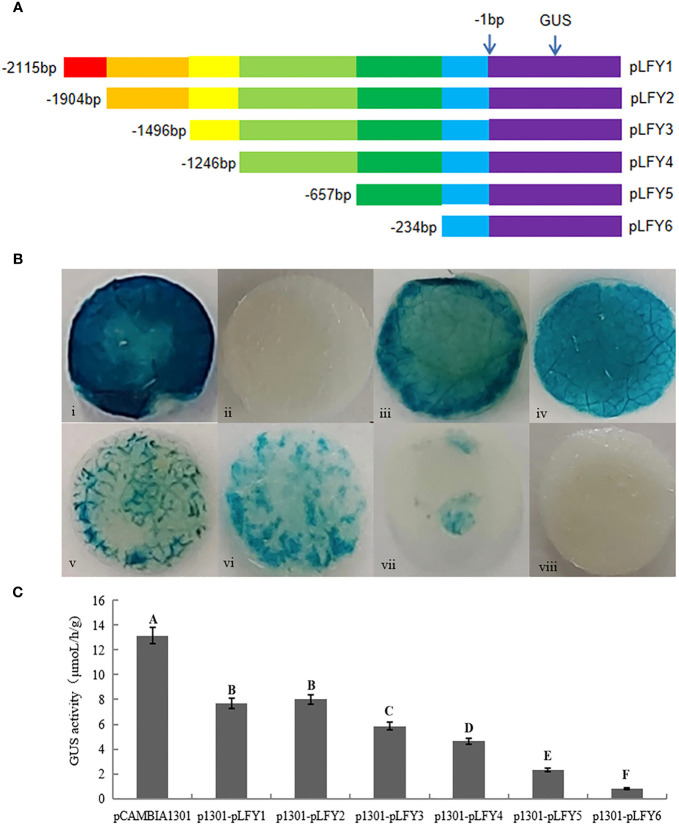
GUS histochemical staining and enzyme activity determination of transiently transformed Nicotiana benthamiana plants with different JmLFY promoter fragments. **(A)** Schematic illustration of different lengths of 5’-deletion primers for JmLFY promoter. **(B)** GUS histochemical staining of positive control (injected Agrobacterium with pCAMBIA1301 empty vector, i), negative control (injected Agrobacterium without vector, ii), p1301- pLFY1 (iii), p1301-pLFY2 (iv), p1301-pLFY3 (v), p1301-pLFY4 (vi), pA1301-pLFY5 (vii), and p1301-pLFY6 (viii). **(C)** GUS enzyme activity determination with different JmLFY promoter fragments. The different uppercase letters above the error bars mean significant difference at α = 0.01.

According to the predicted *cis*-acting element positions on the promoter, the plasmid DNA of pMD19-T-pLFY was extracted and amplified using six primers, which could amplify different lengths of *JmLFY* promoter fragments by designing 5′-deletion primers (**Table 1**, **Figure 1A**). The PCR products were named pLFY1 to pLFY6. The recovered PCR products and pCAMBIA1301 vector were then dual-digested with *Hin*dIII and *Bgl*II rapid endonuclease and ligated using T4 DNA Ligase. The recombined vector was then transformed into *E. coli* DH5α competent cells, and then the positive clones selected by 50 mg/L of kanamycin (Kan) were used for PCR amplification and then sequenced; the correct recombined vectors confirmed by sequencing were named pCAMBIA1301-pLFY1 to pCAMBIA1301-pLFY6, which were abbreviated respectively as p1301-pLFY1 to p1301-pLFY6 hereafter.

### 2.3 Genetic transformation of *JmLFY* promoter

#### 2.3.1 Preparation of *Agrobacterium* EHA105 competent cells

*Agrobacterium* EHA105 strain was cultured on YEP solid medium for 36 h at 28°C, and a single clone was selected and incubated in liquid YEP medium containing 50 mg/L of rifampin (Rif) with 200-rpm shaking frequency at 28°C till OD_600_ reached 0.5–0.6. The liquid was then centrifuged for 5 min at 4,000 *g* at 4°C, the supernatant was discarded, and the precipitate was resuspended in 1 mL of precooled CaCl_2_. The *Agrobacterium* EHA105 competent cells were added in 500 μL of 50% sterilized glycerol, then divided into 100 μL, frozen in liquid nitrogen, and stored in a −80°C freezer.

#### 2.3.2 Recombinant expression vector transformed to *Agrobacterium* EHA105 competent cells

Plasmid DNA extraction of the recombinant vectors (p1301-pLFY1 to p1301-pLFY6) was conducted, mixed with *Agrobacterium* EHA105 competent cells on ice for 30 min, then frozen in liquid nitrogen for 5 min, and transferred to 37°C water bath for 5 min. The transformed products were cultured in liquid YEP medium overnight at 28°C with 200-rpm shaking frequency and then centrifuged for 1 min at 4,000 *g*, and most of the supernatant was discarded. The remaining approximately 100 μL was used for resuspending the precipitate, then cultured on YEP solid medium containing 50 mg/L of Kan and 50 mg/L of Rif, inverted petri dishes, and incubated at 28°C for 2–3 days. The single clone was selected and incubated in a liquid YEP medium containing 50 mg/L of Kan and 50 mg/L of Rif with 200-rpm shaking frequency at 28°C. The solution with propagated transformation products was detected by PCR amplification and 1% agarose gel electrophoresis.

#### 2.3.3 Preparation of infection solution

The verified transformed products were coated on a solid YEP medium containing 50 mg/L of Kan and 50 mg/L of Rif. A single clone was selected and incubated in 50 mL of liquid YEP medium containing 50 mg/L of Kan and 50 mg/L of Rif till OD_600_ reached 0.5–0.6. The liquid was then centrifuged for 10 min at 5,000 *g*, and the supernatant was discarded. The precipitate was resuspended with MS medium [containing 30 g/L of sucrose and 100 μM of acetosyringone (AS)] till OD_600_ reached 0.5–0.6, which was used as an infection solution.

#### 2.3.4 *Agrobacterium*-mediated genetic transformation

The prepared p1301-pLFY1 infection solution was then used for infected leaves of aseptic *Nicotiana benthamiana* seedlings for 30–45 days. The leaves were cut into 0.5–1 cm^2^ and soaked in the infection solution for 8–10 min. The soaked leaves were co-cultured on MS + 1 mg/L 6-BA + 0.1 mg/L NAA at 25°C for 3 days, then washed successively with sterile water containing ceftazidime (Cef) 1,000 mg/L (2 min) and 500 mg/L (1 min), and then washed with sterile water for three times. The sterile leaves were transferred successively on selection medium for callus induction (MS + 1 mg/L 6-BA + 0.1 mg/L NAA + 500 mg/L Cef + 10 mg/L hygromycin (Hyg), 3 weeks), differentiation (MS + 0.5 mg/L 6-BA + 0.05 mg/L NAA + 500 mg/L Cef + 10 mg/L Hyg, 1 week), elongation (MS + 0.2 mg/L 6-BA + 0.02 mg/L NAA + 500 mg/L Cef + 10 mg/L Hyg, 1 week), and rooting (MS + 0.02 mg/L NAA + 500 mg/L Cef + 10 mg/L Hyg, 2 weeks). The rooted tobacco seedlings were transferred into pots and covered with plastic cups with high light transmittance for 2–3 days, and then the covers were removed.

#### 2.3.5 *Agrobacterium*-mediated transient transformation with different treatments of *N. benthamiana*

The prepared p1301-pLFY1 to p1301-pLFY6 infection solutions were transiently transformed into tobacco leaves by injecting using a 1-mL disposable injector. The injected plants were bagged in the dark for 2–3 days and then transferred to light.

Considering that the *JmLFY* promoter contains the light-responsive elements, hormone response elements, and low-temperature responsive elements, the transiently transformed tobacco plants were then treated with photoperiod (16-h light/8-h dark and 8-h light/16-h dark), temperature (4°C and 25°C), and hormone (salicylic acid (SA), ABA, IAA, GA3, and methyl jasmonate (MeJA), with H_2_O as control) spraying to verify the function of the core regulatory region and the *cis*-acting elements of the *JmLFY* promoter. The photoperiod and low-temperature treatments were conducted in transiently transformed tobacco plants with p1301-pLFY1, p1301-pLFY2, p1301-pLFY3, p1301-pLFY4, p1301-pLFY5, and p1301-pLFY6, and the hormone treatments were conducted only in transiently transformed tobacco plants with p1301-pLFY1. After 24 h of treatments, the *GUS* histochemical staining and *GUS* enzyme activity determination were performed.

#### 2.3.6 *GUS* gene expression, staining, and enzyme activity determination in transgenic plants

RNA was extracted from the roots, stems, leaves, and flowers of transgenic plants, which were reverse transcribed into cDNA. *GUS* gene expression in these organs was then detected by qRT-PCR with tobacco *L25* gene as the reference gene, and the primers are listed in **Table 1**. Furthermore, in order to examine the transcriptional activity of *GUS* genes driven by the *JmLFY* promoter in different organs in the transgenic plants, GUS histochemical staining was performed using a *GUS* staining kit (Beijing Coolaber Technology Co., Ltd., Beijing, China).

For the transient transformation tobacco plants, *GUS* histochemical staining and enzyme activity determination (GUS enzymatic activity assay kit, Beijing Coolaber Technology Co., Ltd.) of punched leaves were performed.

### 2.4 Yeast one-hybrid

#### 2.4.1 Prey vector construction

Based on the *JmSOC1* sequence information and the restriction endonuclease *Sma*I site and its flanking sequences of the pGADT7-Rec2 vector, the upstream and downstream primers ADSOC1-F and ADSOC1-R were designed (**Table 1**). The pMD19-T-*JmSOC1* target fragment was then amplified by PCR using plasmid DNA extracted from pMD19-T-JmSOC1 (conserved by Key Laboratory of Forest Tree Genetics and Breeding of Liaoning Province) as a template. Plasmid of yeast vector pGADT7-Rec 2 was extracted and digested using the restriction endonuclease *Sma*I; the products were recovered, purified, and then ligated with pMD19-T-Jm*SOC1* target fragment using NovoRec plus One step PCR Cloning Kit. The ligated products were then transformed into *E. coli* Top10 competent cells, and the bacteria solution was detected by PCR and sequenced to identify the recombinant vector. The correct vector was used as a prey vector for yeast one-hybrid and named pGADT7-SOC1.

#### 2.4.2 Bait vector construction

According to the *JmLFY* promoter sequence and the recognition site of the pHIS2 restriction endonuclease, the *Eco*RI and *Mlu*I restriction sites were introduced at the 5′ end of the upstream and downstream primers of the *JmLFY* promoter. The primers HISpLFY-F and HISpLFY-R with the *Eco*RI and *Mlu*I restriction sites were designed (**Table 1**) and used for *JmLFY* promoter amplification. The amplified *JmLFY* promoter was recovered, purified, dual-digested by *Eco*RI and *Mlu*I, and then ligated to pHIS2 linear vector fragments that were also dual-digested by *Eco*RI and *Mlu*I using T4 DNA Ligase. The ligated products were then transformed into *E. coli* Top10 competent cell, and the bacteria solution was detected by PCR and sequenced to identify the recombinant vector. The correct vector was used as a bait vector for yeast one-hybrid and named pHIS2-LFY.

#### 2.4.3 Point-to-point validation of yeast single hybridization of JmSOC1 and *JmLFY* promoter

The positive control (pGADT7-rec2-p53 and pHIS2-p53), negative control (pGADT7-Rec2 and pHIS2-p53), self-activation assay (pGADT7-Rec2 and pHIS2-LFY), and interaction assay (pGADT7-SOC1 and pHIS2-LFY) were co-transformed into yeast competent cell Y187, and the yeast solution was then coated on DDO medium (SD/-Leu/-Trp) and TDO medium (SD/-His/-Leu/-Trp) with various concentrations of 3-AT (60 mM, 90 mM, 150 mM, and 200 mM). The cultures were placed upside down and incubated at 30°C for 2–4 days.

## 3 Results

### 3.1 Promoter cloning and bioinformatics analysis of JmLFY gene

A 2,170-bp sequence was obtained and compared to the cDNA of *JmLFY* gene and the upstream sequences of *LFY* in *J. regia* published by the National Center for Biotechnology Information (NCBI). A total of 55 bases with 95% similarity at the 3′ end of the obtained sequence overlapped with the 5′ end cDNA of *JmLFY* gene, which indicated that we obtained the upstream sequence of *JmLFY*. Compared with the remaining 2,115 bp to upstream sequences of *LFY* in *J. regia*, over 90% similarity suggested that we obtained the promoter sequence of *JmLFY* gene successfully (SRR24958161, **Figure S1**).

The *LFY* promoter regulatory element analysis showed that various functional *cis*-acting elements exist on *JmLFY* gene promoter, including the core *cis*-acting elements essential for eukaryotic promoters, such as TATA-box, CAAT-box; light-responsive elements, such as Box 4, G-Box, GT1-motif; hormone response elements, such as abscisic acid response element ABRE, MeJA response element CGTCA-motif and TGACG-motif, ethylene-responsive ERE, gibberellin-responsive TATC-box, *cis*-acting element involved in salicylic acid-responsive TCA, and auxin response element TGA, etc.; stress-responsive elements, such as low temperature-responsive *cis*-acting element (LTR), MYB binding site involved in drought inducibility (MBS) and light responsiveness (MRE), *cis*-acting element involved in defense and stress responsiveness (TC-rich repeats), MYB response elements associated with drought, salt and abscisic acid response, MYC associated with drought and abscisic acid response; flowering-related elements, such as the *cis*-acting element POLLEN1LELAT52 that is specifically expressed by pollen, the late pollen gene initiator element GTGANTG10, and the binding site CArG-box motif for flowering-related proteins; and other elements, such as anaerobic-induced regulatory element ARE, *cis*-regulatory element involved in regulation of zein metabolism O2-site, damage response element WRE3, etc.; and some elements of unknown function (**Table S1**, **Figure S1**). These results suggested that the expression of *LFY* gene may be regulated by several factors.

### 3.2 *Agrobacterium*-mediated genetic transformation of *N. benthamiana* and validation

#### 3.2.1 *Agrobacterium*-mediated genetic transformation of *N. benthamiana*

**Figure 2:**
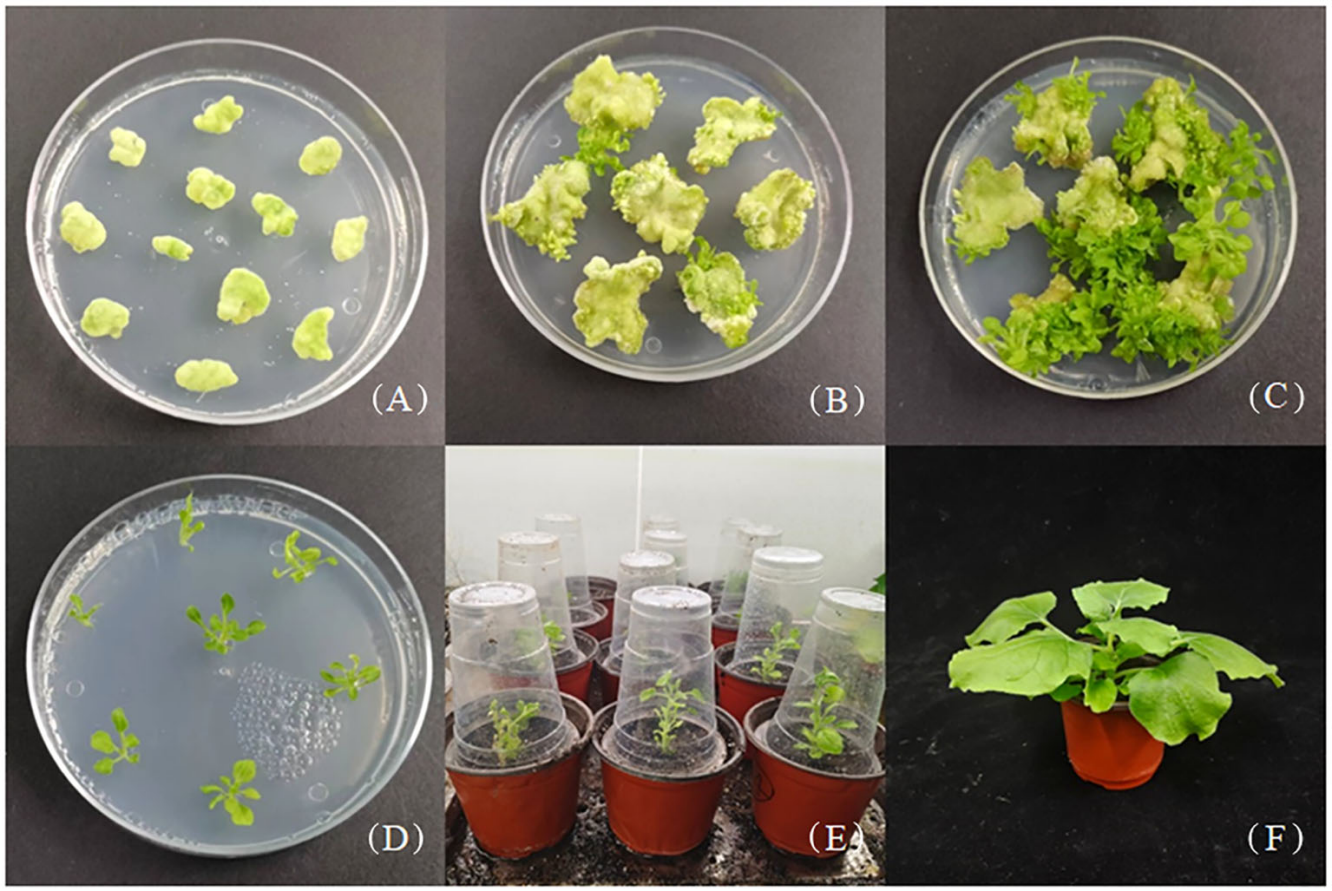
Genetic transformation of Nicotiana benthamiana with p1301-pLFY1. **(A)** Callus induction. **(B, C)** Callus differentiation and adventitious shoot elongation. **(D)** Rooting of adventitious shoots. **(E)** Transplanting. **(F)** Transgenic plant.

To identify the core regulatory regions of the *JmLFY* promoter, a total six of expression vectors (p1301-pLFY1 to p1301-pLFY6) with different lengths of 5′-deletion fragments were constructed successfully. The expression vector p1301-pLFY1 was transformed into *N. benthamiana*, and regenerated plants were obtained via callus induction, differentiation, elongation, rooting, and acclimatization (**Figure 2**), and six transgenic tobacco plants were determined by PCR with *GUS* gene universal primer and *pLFY 1*-specific primer (**Figure S2**).

#### 3.2.2 *GUS* histochemical staining and gene expression in transgenic *N. benthamiana*

**Figure 3:**
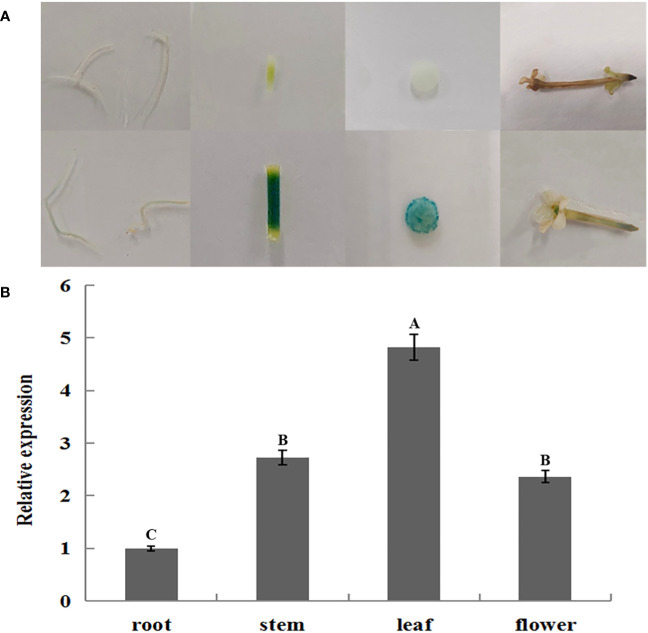
GUS histochemical staining and qRT-PCR analysis of GUS gene in different organs of transgenic Nicotiana benthamiana. **(A)** Root, stem, leaf, and flower of wild type (upper) and transgenic (lower) tobacco. **(B)** qRT-PCR analysis of GUS gene in root, stem, leaf, and flower of transgenic N. benthamiana. The different uppercase letters above the error bars mean significant difference at α = 0.01.

*GUS* histochemical staining in different organs of transgenic tobacco showed that *JmLFY* gene promoter drove *GUS* gene transcriptional activity in all detected organs, which stained the deepest in the leaf and the slightest in the root (**Figure 3A**). qRT-PCR was also performed on roots, stems, leaves, and flowers of the transgenic tobacco, which showed similar results as GUS histochemical staining. The expression of *GUS* gene was the highest in the leaf of transgenic tobacco and the lowest in the root (**Figure 3B**).

### 3.3 Expression of *GUS* gene driven by different *JmLFY* promoter fragments in transient transformed tobacco plants

GUS staining showed that the positive control (injected *Agrobacterium* with pCAMBIA1301 empty vector) was stained the deepest, and the leaves of negative control (injected *Agrobacterium* without vector) were not stained. The other leaves from transiently transformed plants were stained except for p1301-pLFY6, of which p1301-pLFY1 and p1301-pLFY2 were stained deeply, followed by p1301-pLFY3 and p1301-pLFY 4, and p1301-pLFY5 was stained slightly (**Figure 1B**).

GUS enzyme activity determination confirmed the histochemical staining results further (**Figure 1C**), which showed the highest activity of positive control, followed by p1301-pLFY2, p1301-pLFY1, p1301-pLFY3, p1301-pLFY4, p1301-pLFY5, and p1301-pLFY6 successively.

### 3.4 Effects of different treatments on activity of *GUS* gene driven by different *JmLFY* promoter fragments in transient transformed tobacco plants

#### 3.4.1 Effects of photoperiod on *GUS* gene activity

**Figure 4:**
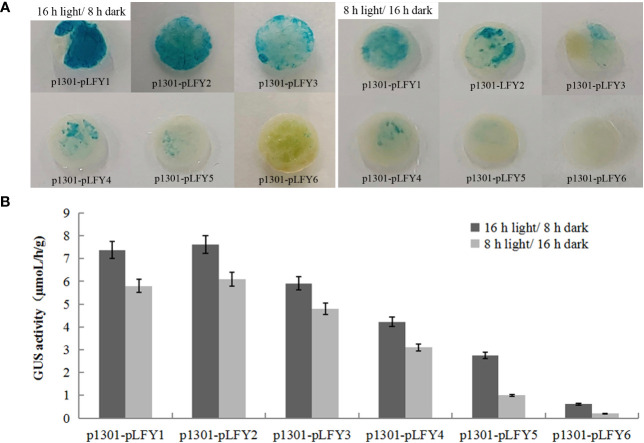
GUS histochemical staining **(A)** and enzyme activity determination **(B)** of transient transformed *Nicotiana benthamiana* plants with different JmLFY promoter fragments under long (16-h light/8-h dark) and short (8-h light/16-h dark) photoperiod.

*GUS* histochemical staining of transiently transformed plants treated by 2 days of long photoperiod (16-h light/8-h dark) and short photoperiod (8-h light/16-h dark) showed that transiently transformed plants with different *JmLFY* promoter fragments were stained in different levels, which decrease progressively from p1301-pLFY1 to p1301-pLFY6 (**Figure 4A**). However, all the leaves from plants treated by a long photoperiod were stained more strongly than those treated by a short photoperiod (**Figure 4A**). *GUS* enzyme activity determination showed similar results to *GUS* histochemical staining (**Figure 4B**).

#### 3.4.2 Effects of temperature on *GUS* gene activity

**Figure 5:**
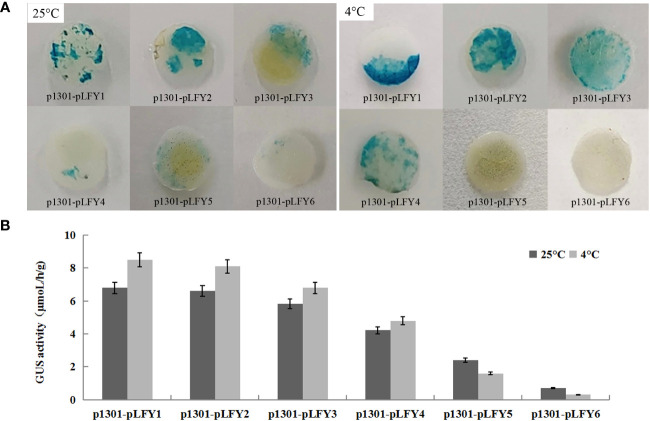
GUS histochemical staining **(A)** and enzyme activity determination **(B)** of transiently transformed *Nicotiana benthamiana* plants with different JmLFY promoter fragments under 25°C and 4°C.

*GUS* histochemical staining of transiently transformed plants treated at normal temperature (25°C) and low temperature (4°C) showed that transiently transformed plants with different *JmLFY* promoter fragments were stained at different levels (**Figure 5A**). However, leaves from plants treated with low temperatures were stained more strongly than those treated with normal temperatures except for p1301-pLFY5 and p1301-pLFY6 (**Figure 5A**). *GUS* enzyme activity determination showed similar results to *GUS* histochemical staining (**Figure 5B**).

#### 3.4.3 Effects of hormone spray on *GUS* gene activity

**Figure 6:**
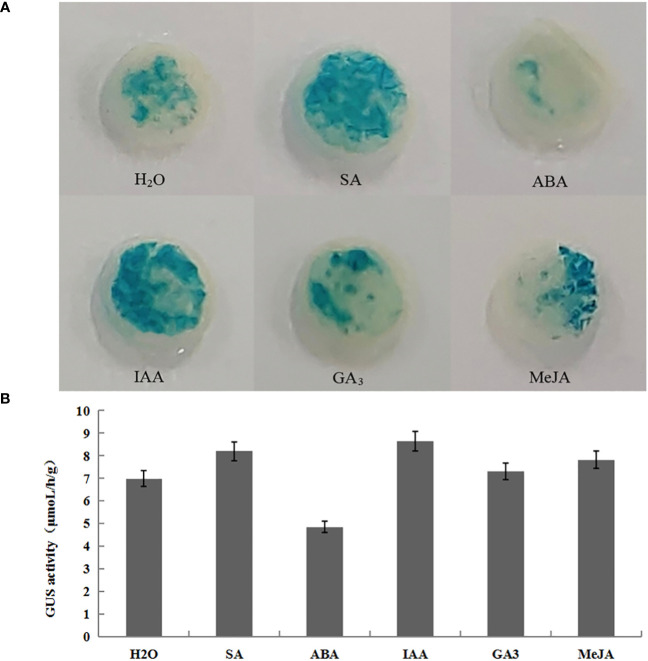
GUS histochemical staining **(A)** and enzyme activity determination **(B)** of transiently transformed Nicotiana benthamiana plants with p1301-pLFY1 by spraying different hormones.

*GUS* histochemical staining and enzyme activity determination of transiently transformed plants treated by hormones showed that the treated transiently transformed plants with p1301-pLFY1 had higher activities than those treated by H_2_O (control), except for ABA, which showed lower activities than control (**Figure 6**). These results indicated that the p1301-pLFY1 promoter fragment responded to the induction of SA, IAA, GA3, and MeJA, and the treatments of SA, IAA, GA3, and MeJA increased the activities of *JmLFY1* promoter.

### 3.5 Point-to-point validation by yeast one-hybrid of JmSOC1 and *JmLFY* promoter

**Figure 7:**
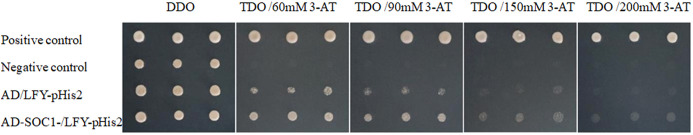
Point-to-point verification of yeast one-hybrid for co-transforming yeast plasmid. DDO and TDO mean Synthetic Dropout Media (SD) with Double Dropout Supplements (DDO, SD/-Leu/-Trp) and Triple Dropout Supplements (TDO, SD/-His/-Leu/-Trp).

Point-to-point validation by yeast one-hybrid of JMSOC1 and the *JmLFY* promoter showed that all co-transformed yeast plasmid grew well on the DDO medium, which suggested that the co-transformations are successful and without obvious toxic effect on the Y187 yeast strain (**Figure 7**). Self-activation decreased followed by increasing concentration of 3-AT on TDO medium, which was inhibited completely when 3-AT concentration was over 90 mM. The strains of positive control (pGADT7-Rec2 + pHIS2-p53) and the interaction assay (pGADT7-Rec2 + pHIS2-LFY) grew well on TDO medium supplied with 60 mM and 90 mM, which indicated the interaction between JmSOC1 and the *JmLFY* promoter.

## 4 Discussion

*J. mandshurica* is a monoecious species with heterodichogamous characteristics. The non-synchronous flowering and development of male and female flowers limited the mating and the yield and quality of fruits of the species. *LFY* has a core position in the flowering regulatory networks in plants, and the overexpression of *JmLFY* gene has been confirmed to promote flowering approximately 7 days in advance and supplement the phenotypic defects of *lfy* mutant partially in *Arabidopsis* (Cai et al., 2023). In this study, we cloned the *JmLFY* promoter of 2,115 bp in length, which has more than 90% homologous sequence aligned with the *J. regia* promoter. *GUS* histochemical staining and expression analysis showed that the *JmLFY* promoter drove the expression of *GUS* gene in the leaves, stems, flowers, and roots of transgenic tobacco plants, which indicated that the *LFY* promoter had driving activity. Similar results were also reported in *Populus tomentosa LFY* promoter (Li et al., 2012a).

Bioinformatics analysis of the obtained sequence revealed that the *JmLFY* promoter contains the core *cis*-acting element essential to the eukaryotic promoter, such as TATA-box and CAAT-box, which are consistent with the basic structural characteristics of the promoter (Molina and Grotewold, 2005). In addition, some flowering-related elements, such as POLLEN1LELAT52, GTGANTG10, and CArG-box motif, were also reported in the *LFY* promoter of *Dimocarpus longan* (Xu et al., 2011).

To further determine the *cis*-elements and their function on the *JmLFY* promoter, several promoter fragments with different lengths of 5′-deletion were cloned and transformed transiently into tobacco plants. Decreased *GUS* activities followed by the reduction of *JmLFY* promoter sequences (**Figures 4**–**6**) indicated that the driving capacities of promoter fragments decreased by reduction of length, which might be caused by the decreasing numbers of responsive elements in the *JmLFY* promoter fragments. The higher activity of p1301-pLFY2 than p1301-pLFY1 suggested that a negative regulation region might exist in −1,904 bp to −2,115 bp of the *JmLFY* promoter, which needs to be studied further.

Light-responsive elements, such as Box 4, G-Box, and GT1-motif, which have been reported in *LFY* promoters in *J. regia* and Carya cathayensis (Sun et al., 2017), were also detected on the *JmLFY* promoter, which indicated that the promoters might be induced by light. In this study, long photoperiod (16-h light/8-h dark) enhanced the driving capacity of all the *JmLFY* promoters and confirmed that the light-responsive elements on the *JmLFY* promoter are more sensitive to the long photoperiod than the short photoperiod. The differences in *GUS* staining and enzyme activity among different-length promoter fragments might be related to the deletion of light-responsive elements in different promoter fragments.

Low temperature enhanced the driving capacity of all the *JmLFY* promoters except for p1301-pLFY5 and p1301-pLFY6, which showed higher activities under normal temperature than low temperature. However, the location of low temperature-responsive *cis*-acting element (LTR, −2,016 bp in **Table S1**) was contained by all the promoter fragments, suggesting that other sites that responded to low temperature might exist in the −657-bp to −2,115-bp fragments of the *JmLFY* promoter. In C. cathayensis, the *LFY* promoter expression also increased after being treated with low temperature and light (Li, 2012b), which is consistent with our research. However, the specific mechanism that low temperature and long photoperiod promoted the expression of *LFY* promoter was still unknown, which is worth studying further.

In *Arabidopsis*, some hormone response elements existed on *LFY* gene promoter, which responded to the induction of auxin (Nobutoshi et al., 2016). Similarly, a series of hormone response elements such as ABRE, MeJA response element, CGTCA-motif, TGACG-motif, ERE, P-box, TCA-element, and TGA-element (**Table S1**) were also detected on the *JmLFY* promoter. Exogenous spraying of SA, IAA, GA3, and MeJA to the transiently transformed plants with p1301-pLFY1 increased *GUS* activities than that treated by H_2_O (control), and exogenous spraying of ABA decreased *GUS* activities (**Figure 6**), which indicated the positive regulation of SA, IAA, GA3, and MeJA and the negative regulation of ABA. In *N. benthamiana* and rice, *Ferredoxin 1* (*FD1*) promoter activity was repressed when applying exogenous ABA. The result was explained as that the accumulation of ABA stimulates the expression of *ABI5* (ABSCISIC ACID-INSENSITIVE 5), an ABA-responsive transcriptional factor, which negatively regulates *FD1* by binding to ABRE motifs in the *NbFD1* promoter (Cui et al., 2021). Thus, we speculated that the inhibition of *JmLFY* promoter activity by ABA might also be due to a similar causation because two ARBEs existed on the *JmLFY* promoter. Currently, both positive and negative effects of endogenous or exogenous ABA on plant flowering have been reported. For example, ABI4 and ABI5 activated transcription of the flowering repressor gene *FLOWERING LOCUS C* (*FLC*) by binding the *FLC* promoter directly and then repressed the floral transition (Wang et al., 2013; Xiong et al., 2019). Exogenous application of ABA delayed flowering in plants (Wang et al., 2013). These results indicate the negative effects of ABA on floral transition. On the contrary, endogenous ABA upregulated *FLOWERING LOCUS T* (*FT*) expression in *Arabidopsis thaliana*, and root application of exogenous ABA in soil accelerated *Arabidopsis* flowering (Riboni et al., 2016), which indicated the positive effects of ABA on floral transition. In *J. mandshurica*, our previous study showed that the endogenous ABA content increased gradually with the differentiation of flower buds (Qin et al., 2022); however, no further studies were conducted. Thus, it is necessary to study the specific effects of ABA on the flowering of *J. mandshurica*.

It was found that the conserved binding domain of MADS-box on the SOC1 could specifically bind to the DNA sequence containing CArG-box, thus regulating the expression of the downstream target *LFY* gene (Kaufmann et al., 2005). However, a missense mutation in the MADS box of SOC1 could not bind to the *LFY* promoter and then suppressed the flowering promotion function. Similarly, the *LFY* promoter without CArG-box could not be bound with MADS-box on the SOC1 in *Gossypium hirsutum* (Li et al., 2013). In this study, the bioinformatics analysis of the *JmLFY* promoter showed that two CArG-box domains (CAATATATAG, −1,868 bp, and CCTTTATAGG, −1,919 bp; **Table S1**, **Figure S1**) were present on the promoter sequence. In order to prove the interaction between JmSOC1 and *JmLFY*, the pGADT7-Rec2-JmSOC1 prey vector and the pHIS2-pLFY bait vector were constructed for yeast one-hybrid point-to-point verification. The results showed that JmSOC1 interacted with *JmLFY* gene promoter. However, whether the JmSOC1 can affect the transcriptional activation of the *JmLFY* promoter *in vivo* needs to be studied in the future. Similar results were also confirmed by the CHIP test of SOC1 and *LFY* promoter in *Arabidopsis* (Lee et al., 2008; Liu et al., 2008).

## Data availability statement

The original contributions presented in the study are included in the article/**Supplementary Material**. Further inquiries can be directed to the corresponding author.

## Author contributions

LZ and CL contributed to the conception and design of the study. JF, TD, MZ, and JW organized the database. JF and TD performed the statistical analysis. LZ and JF wrote the first draft of the manuscript. CL revised and edited the manuscript. All authors contributed to the article and approved the submitted version.
