# Supplementary material for: Promoter cloning and activities analysis of JmLFY, a key gene for flowering in Juglans mandshurica
Source: Front Plant Sci. 2023 Oct 12;14:1243030. doi: 10.3389/fpls.2023.1243030 (PMC10602732; doi:10.3389/fpls.2023.1243030)
Supplement: Supplementary file 1 [file DataSheet_1.docx]

Supplementary Material

Promoter cloning and functional analysis of *JmLFY*, a key gene for flowering in *Juglans mandshurica*

Lijie Zhang^1,2^, Jingqi Fu^1,2^, Tianyi Dong^1,2^, Mengmeng Zhang^1,2^, Jingwen Wu^1,2^,Chunping Liu^1,2,3*^

*** Correspondence:** Chunping Liu [liuchunping2019@syau.edu.cn](mailto:liuchunping2019@syau.edu.cn)

# Supplementary Figures and Tables

## Supplementary Figures


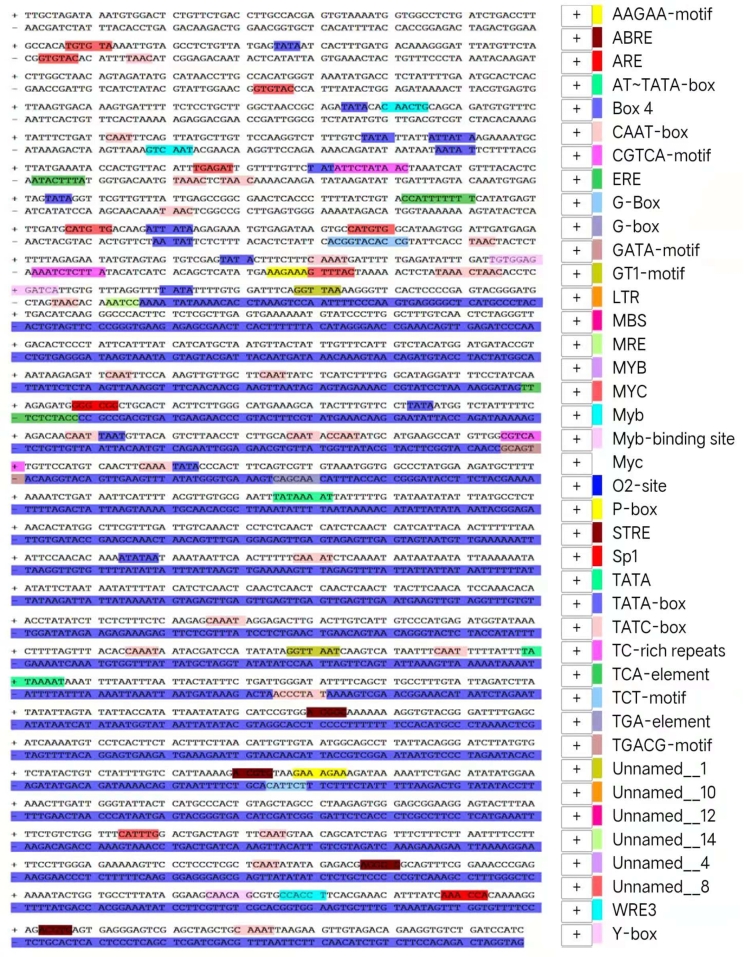


**Supplementary Figure 1.** Promoter sequence of *JmLFY* gene in *Juglans mandshurica* and the cis-acting elements analysis


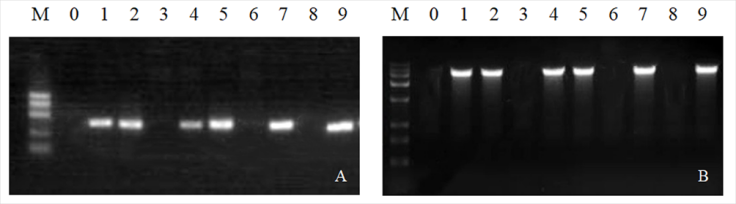


**Supplementary Figure 2.** PCR identification of transgenic *Nicotiana benthamiana*. M: DL2000 marker; 0: Control; (A) *GUS* gene universal primer amplification; (B) pLFY1 specific primer amplification.

## Supplementary Table

**Supplementary Table 1.** Distribution of cis-acting elements of *JmLFY* promoter in *Juglans mandshurica*

| **Site name** | **Location^1^** | **Motif sequence** | **Function** | **Quantity** |
| --- | --- | --- | --- | --- |
| ABRE | +1780,+2103 | ACGTG | abscisic acid response element | 2 |
| ARE | -1898,+2088 | AAACCA | anaerobic inducible regulatory element | 2 |
| Box 4 | +989,-1630 | ATTAAT | part of a conserved DNA module involved in light responsiveness | 2 |
| CAAT-box | -85,+292,… | CAAT/ CAAAT | common cis-acting element in promoter and enhancer regions | 32 |
| CArG-box motif | -1868,-1919 | CAATATATAG/ CCTTTATAGG | the binding site of flowering associated proteins | 2 |
| CGTCA-motif | +1046 | CGTCA | MeJA(methyl jasmonate) hormone response element | 1 |
| ERE | -352,-1118 | ATTTCATA/  ATTTTAAA | ethylene response element | 2 |
| G-Box | -532,-1779,  -2102 | TCCACATGGCA/  CACGTC | cis-acting regulatory element involved in light responsiveness | 3 |
| GATA-motif | -902,-1957 | GATAGGA/  AAGGATAAGG | part of a light responsive element | 2 |
| GT1-motif | +668,+150,  -1004 | GGTTAA/  GGTTAAT | light responsive element | 3 |
| GTGANTG10 | -57,-1435,… | GTGA | late pollen gene promoter | 6 |
| LTR | -2016 | CCGAAA | low temperature responsive cis-acting element | 1 |
| MBS | +260 | CAACTG | MYB binding site involved in drought-inducibility | 1 |
| MRE | -642 | AACCTAA | MYB binding site involved in light responsiveness | 1 |
| MYB | +2056 | CAACAG | associated with drought, salt, and abscisic acid responses | 2 |
| MYC | -73,+497,… | CATGTG | associated with drought and abscisic acid response | 8 |
| O2-site | -1055 | GATGACATGG | cis-acting regulatory element involved in zein metabolism regulation | 1 |
| P-box | -2094 | CCTTTTG | gibberellin-responsive element | 1 |
| POLLEN1LELAT52 | -136, -195,… | AGAAA | cis-acting elements of pollen specific expression | 5 |
| Sp1 | +918 | GGGCGG | light responsive element | 1 |
| TATA-box | +105,+336,… | TATA/ TATAA/ ATTATA/TATAAAA | transcription start site-30 core promoter elements | 56 |
| TATC-box | -1575 | TATCCCA | cis-acting element involved in gibberellin-responsiveness | 1 |
| TC-rich repeats | +393,-562 | ATTCTCTAAC | cis-acting element involved in defense and stress responsiveness | 2 |
| TCA-element | +472,-909 | CCATCTTTTT | cis-acting element involved in salicylic acid responsiveness | 2 |
| TGA-element | -1085 | AACGAC | auxin-responsive element | 1 |
| TGACG-motif | -1046 | TGACG | cis-acting regulatory element involved in the MeJA-responsiveness | 1 |
| WRE3 | +2066 | CCACCT | damage response element | 1 |

^1^ + and - indicate forward and reverse strands, and ... indicates omitted sites.
